# Does Forum Theater Help Reduce Gender Inequalities and Violence? Findings From Nepal

**DOI:** 10.1177/0886260521997457

**Published:** 2021-03-04

**Authors:** Pranab Dahal, Sunil Kumar Joshi, Katarina Swahnberg

**Affiliations:** 1 Linnaeus University, Kalmar, Sweden; 2 Kathmandu Medical College, Kathmandu, Nepal

**Keywords:** cultural contexts, domestic violence, intervention/treatment, prevention, sexual assault

## Abstract

Gender inequality and violence against women are present in every society and culture around the world. The intensities vary, however, based on the local guiding norms and established belief systems. The society of Nepal is centered on traditional belief systems of gender roles and responsibilities, providing greater male supremacy and subordination for the females. This has led to the development and extensive practices of social gender hierarchal systems, producing several inequalities and violence toward women. This study has utilized Forum Theater interventions as a method of raising awareness in 10 villages in eastern Nepal. The study aimed to understand the perception and changes in the community and individuals from the interactive Forum Theater performances on pertinent local gender issues. We conducted 6 focus group discussions and 30 individual interviews with male and female participants exposed to the interventions. The data analysis utilized the constructivist grounded theory methodology. The study finds that exposure and interactive participation in the Forum Theater provide the audience with knowledge, develop empathy toward the victim, and motivate them to change the situation of inequality, abuse, and violence using dialogues and negotiations. The study describes how participation in Forum Theater has increased individual’s ability for negotiating changes. The engagement by the audience in community discussions and replication of efforts in one of the intervention sites show the level of preparedness and ownership among the targeted communities. The study shows the methodological aspects of the planning and performance of the Forum Theater and recommends further exploration of the use of Forum Theater in raising awareness.

## Introduction

In the remote Kerabari village of Morang district in eastern Nepal, a crowd has gathered, and it is interrogating a man who looks terrified. More mass gathers up as it is a weekly market day. The arguments heat up, the topic of discussion is barely heard from the distance but the gestures of the agitated crowd with pointed fingers and closed fists confirm something is seriously wrong. Suddenly, one of the middle-aged women from the crowd grabs one of the slippers from her foot and swiftly raises it high above her shoulder, and lean forward to charge the petrified man. He flees tearing the crowd apart and vanishes into the thin air like the disappearing dust which had risen while he was running away.

The narration above is a description of the Forum Theater performance and the only actor in the scene was the fleeing man who was performing the role of trafficker interrogated by the local community turned “spect-actors.” The emotions involved were sincere, the rising rage was genuine, and the event was close to reality.

The use of theater in scientific research is scarce; nonetheless, this amalgamation of scientific research and performing arts provides opportunities to recognize and investigate social actions and oppressions (Barone & Eisner, 2012; Sullivan, 2010). The use of theater can be an enlightening experience for the participating audience as it primarily focuses on human experiences, emotions, and interactions that occur in their daily lives (Sullivan, 2010). Augusto Boal, the pioneer of the Forum Theater, identifies that theater has the potential to bring social transformation as it enriches knowledge and encourages audiences to act for change (Boal, 1992). Borrowing the ideas from Pablo Freire, who developed learning methods based on dialogue, emphasized the development of critical consciousness, which he coined as “conscientization” which focuses on recognizing and evaluating existing power structures (Friere, 1970). “Praxis” is another concept that relates to the process of embodied learning through critical reflection and further—the concept of “decodification” stresses identifying situations until one completely gets immersed in the situation. Boal utilized these concepts to develop the theater of the oppressed on the basis of the critical reflection of embodied learning and immersion to the situation, but he does not define oppression rather encourages one to find it for and by themselves. Boal (1994) summarizes the focus of Forum Theater as a method for not only trying to solve a particular problem but also for making people aware of the problem and the possibility of finding different ways for dealing with a problem. Forum Theater encapsulates the idea that learning is a result of the interaction, dialogue, and communication. Forum Theater itself does not eliminate oppression but exposes the audience to understand power relations that produce oppressors and the oppressed (Feldhendler, 1994). The participatory Forum Theater allows audiences to rehearse reality in a safe environment, enriching their physical, emotional, and intellectual understanding (Smagorinsky, 1999). Forum Theater methodology is useful, with its dialogue, and enables the audience to dislodge deep-seated concerns, thereby prioritizing sensitivity and respect for ending oppressions (Sullivan & Parras, 2008).

Gender has evolved as a pivot for organized social relations, and the inequalities for women have expanded as a natural occurrence (Benhabib et al., 1995; Ridgeway, 2009). Violence consequences due to gender-based inequalities and the unequal power relationship between men and women which are practiced variously as a normal occur in the daily lives (Connell, 2014; DFID, 2012; Dillon et al., 2013; Yodanis, 2004). Furthermore, this universal presence of gender systems, with the emergence of social structures valuing male superiority, has provided a greater influence on human and social interactions (Gayle, 1975). Thus, violence against women arises not only as an issue affecting individuals but also as a political concern, as well as a means of controlling women under given social structures and ideologies (Ahmad & Stewart, 2004; Carter, 2015; Dobash & Dobash, 1979; Hunnicutt, 2009).

Close to 80% of the human rights victims in Nepal are women, and among them, the cases of violence against women are more than half (INSEC, 2020). Women in Nepal face problems of violence, dowry-related-violence, witchcraft accusation, forced prostitution, and trafficking, which are rooted in the practices of gender-based inequality (Standing et al., 2006; The Asia Foundation, 2010). This inequality arises from unequal sociocultural norms, religious ideologies, and strict adherence to the traditionally defined gendered roles and responsibilities (UNFPA, 2008). Nepal is a patriarchal society, with greater pressures on women to follow strict social conventions and norms, where women constantly face sanctions and discrimination (Boyce & Coyle, 2013). Despite legal reforms, women in Nepal continue to face inequalities due to hegemonic norms embedded in the sociocultural institutions exhibited in daily interactions (Leve, 2007).

Previous studies have identified that gender-based inequalities and violence require interventions that raise questions and challenge established gender norms; critically evaluate the behaviors that produce and sustain inequalities; identify reasons for maintaining power relationships; and encourage active participation of males in sustaining changes produced by the targeted interventions (Dahal et al., 2019; Fulu et al., 2014; Heise & Manji, 2015; Jewkes et al., 2014). Studies using Forum Theater on gender violence prevention has shown that its use has enabled the participants to widen their knowledge with increased optimism for change and develop lower tolerance toward oppressive situations (Mitchell & Freitag, 2011). Similarly, the use of Forum Theater has been tested to reduce victim-blaming, altered the outcomes of violence, sensitized the audience on gender roles and identities, and provided information to resolve conflicts (Conrad, 2004; Lev-Aladgem & First, 2004; Mitchell & Freitag, 2011; Sliep et al., 2004; Wang, 2010).

The use of Forum Theater for academic research is a new concept in Nepal. The existing use of Forum Theater is largely limited to activism, awareness-raising, and civil society issues. To the best of the authors’ knowledge, this study is the first of its kind in Nepal as it has reached a wider audience and geographic coverage of 10 villages in the Morang district of eastern Nepal. This study is part of a larger intervention-comparison project to promote gender equality, reduce violence against women, and increase awareness of sex trafficking. This study aims to increase the understanding of Forum Theater interventions in reducing gender-based inequality, violence, and sex trafficking in Nepal. The study adds to the existing body of knowledge on how Forum Theater is perceived by the audience, and how it has motivated the audience to engage in individual and collective actions. This study is based on longitudinal findings allowing the study participants to absorb the learning acquired from the Forum Theater interventions. Moreover, the methodological explanation of the used interventions can be useful for practitioners and researchers.

## Forum Theater Interventions

The Forum Theater performances for this study were developed by Actors’ Studio, Nepal, Forum Theater’s partner for this study. Artistic and theatrical autonomy was provided to the partner for planning the interventions. The first author was involved with community coordination, provided inputs for the script development and its finalization, and supported the partner with logistical and technical help during interventions. The second and last author provided the supervision. The script for the Forum Theater interventions was developed after an initial site visit. This helped to add details of local daily life, language, sociocultural aspects, music, and lifestyles to developing stories for the Forum Theater. The site visits also helped increase familiarity with the community and develop networks with the local stakeholders.

Three rounds of Forum Theater interventions were organized in 10 different intervention villages for a year (January 2017, May 2017, and January 2018), whereas 10 comparison villages received no interventions. The intervention and comparison villages were selected randomly based on the study plan and design adapted by the project. The Forum Theater interventions were performed in 10 villages in the Morang district: *Amardaha, Babiya Birta, Bahuni, Dangihat, Govidapur, Jhurkiya, Kerabari, Keraun, Tetariya,* and *Yangshila*.

The first script for the Forum Theater, *Ramri Keti*, translates into a beautiful girl; it tells the story of a girl who discontinues her school education to get married. The girl later faces physical and verbal abuse from her husband who develops a habit of gambling and drinking, causing financial instability for the new family. The husband throws the girl out of the house and both her family and the in-laws disown her, as both families were against the marriage. The girl, losing her social and financial ties, wants to leave the village. She finds a female agent who is sympathetic to her situation and promises her a decent job in India. As the story unfolds, it is later identified that she had been tricked into sex trafficking but is rescued at the border checkpoint in Nepal. The storyline for the Forum Theater was based on physical and emotional violence, alcohol and gambling induced violence, lack of support for female victims, and sex trafficking.

The subjects for the second and the third scripts for Forum Theater were developed in consultation with the intervention population. The discussions with the study participants during the focus group discussions and individual interviews for this study helped to identify issues, subjects, and concerns of importance to the local community. The second Forum Theater, *Mana Maya Harayin*, which translates into the Case of the Missing Mana Maya, was developed as an investigative Forum Theater. The story is about *Mana Maya*, a shy young girl who is bullied in school by her male classmates. Her mother is dead and she lives with her alcoholic father. After school each day, she must serve food and drinks to her father’s drinking and gambling friends at home. The girl goes missing from the village after she discovers that a photo of her, which had been edited into a naked image, went viral on social media. The subjects for the Forum Theater were bullying and eve-teasing, sexual harassment, alcohol, gambling, and cybercrime. The final Forum Theater script, Delhi to Dubai, shows the journey of a young woman. The story revolves around a fatherless young woman, solely responsible for a sick mother and a younger brother. The family has amassed a large debt with the mother's medical treatment and the young son's education. The young girl, determined to pay off the debts, decides to quit school and look for foreign employment. The employment agent promises her a job in Dubai. The girl finds herself tricked into sexual slavery in a brothel in Delhi, India but she manages to escape. The storyline for the Forum Theater revolves around social hierarchy, poverty, sex trafficking, life in a brothel, witchcraft accusation, and reintegration challenges faced by sex trafficking survivors.

The scripted Forum Theater shows lasted for an average of 45 minutes to an hour, with live music, song, and dance performances. The joker informed the audience before the show, during the game session, that they could stop the show where they deemed it appropriate to have an exchange with the oppressed or bystanders in the scenes. The audience stopped the play when they felt the story was leading to unacceptable suffering from unjust treatment. The audience participation and exchange in Forum Theater depended on the depth of discussion and engagement. The entire Forum Theater performance usually lasted for 3 hours. The police and members of the community groups (mother’s group, women’s group, female community health volunteers, youth group, etc.), and local teachers participated in the Forum Theater together with the study respondents.

## Method

Honebein (1996) describes the constructivism philosophical paradigm as an approach where individual constructs understanding and knowledge of the world by experience and through reflection on those experiences. This approach was suitable for this study as it helped to construct meaning based on the understanding of both researchers and the participants. The study team believed that reality is subjective and hence acknowledged the existence of multiple realities for which they relied on interactions and experience sharing to develop and co-construct perceived truths.

### Study Participants

A total of 36 participants, with 16 males and 20 females, were included in the focus group discussions. All focus group study participants belonged to the same ethnic community, *Tharu.* Most of the participants for the focus group were 20-29 years old. Only one female participant was unmarried, while a single married male was involved in the discussions. All participants were literate with four males completing a bachelor level of education. Seven female participants had education below 12 years of education. A nuclear family, consisting of a husband-wife and their children, was the major family type identified in both the male and female discussion groups. A total of 30 individuals, 17 females and 13 males, were interviewed immediately after the Forum Theater performances. The individual interview participants belonged to different ethnic and caste backgrounds including upper caste (*Brahmin, Chettri*), *Dalit, Rajbanshi, Dhimal, Tharu, Janajati*, and *Madheshi*. The participants for the interview majorly fell in the age group of 21-45 years.

The selection of participants used a purposive sampling method, and the participation was voluntary. The study participants were recruited with the help of local field staff for the interviews and participants themselves also helped to reach other participants for the focus groups. There were two inclusion criteria set for the participants. First, the participants had to be part of the baseline population of the larger study. Second, they had to witness and/or participate in the Forum Theater interventions.

### Data Collection

The study is based on 6 focus group discussions, 3 each with separate male and female groups and 30 individual interviews, a single interview with a participant from each of the 10 Forum Theater intervention villages. An earlier study suggests that a theatrical performance, which is authentic and relates to the target community, receives greater engagement and empathy from the participating audience (Rohd, 1998). The first author, together with the theater partner team, involving the director, scriptwriter, and production manager visited a few villages in Morang for an appraisal study to develop the content for the upcoming Forum Theater interventions. The team conducted formal and informal interviews with the locals such as representatives of the women’s/mother’s group, teacher, police, leaders, and health professionals, to gather information on the situation of gender inequality, types of local violence, and severity, coping mechanisms, and efforts required to establish equality.

The discussion with the male and the female groups used a multistage focus group method (Hummelvoll, 2008), in which the same group explored and discussed several issues of importance 3 times over a year. The use of a multistage focus group was useful for this study as it strengthened the relationship with the study participants providing them comfort to speak up on their individual experiences on the sensitive issue. Moreover, it also helped to gain clarity on the issues and facilitated data analysis through constant engagements. The use of the focus group discussion helped to develop interactions and discussions about the participants’ understanding of the interventions. Earlier research has identified that focus group discussion helps to develop a relationship with the participants, contributing to the collection of first-hand information on the perceived impact of the used intervention (Hansen et al., 1998). The first author moderated all focus group sessions and a local field staff helped with notetaking, and the last author observed the discussions. The focus group discussions lasted for more than an hour for each session.

All focus group discussions and interviews were audio-recorded. The first and last authors developed the guides for focus group discussions and individual interviews. It included questions about perceptions on the intervention method, the knowledge gained from the shows, gender practices at the community level, and the impact of the shows. The participants for the interviews included both active and passive Forum Theater audiences. The individual interviews allowed us to probe into several issues, making the information collection more rigorous. The choice of the interview method helped to increase understanding and explore the perception of the study participants. The use of structured and open-ended questions helped to gather consistent information on diverse perspectives shared by the participants. The interviews ranged from 10 to 20 minutes and interview notes were also taken.

### Data Analysis

The recordings from the focus group discussions and individual interviews were translated verbatim into English by the first author. The data analysis used Constructivist Grounded Theory (Chamraz, 2010), where descriptive themes were developed after a line-by-line analysis of the transcripts. The line-by-line analysis conducted by the first author provided a summary of the data, and the descriptive themes developed together with the last author helped further to develop emerging analytical categories. The initial coding, focused coding, developed memos, and field/interview notes allowed the researchers to scrutinize the data to interpret socially constructed knowledge. Using this method helped us to develop a theoretical understanding of how participation and/or observation of Forum Theater instill individual change and community engagement in fighting against the perceived odds. The focus group discussions and interviews themselves became the site of creating knowledge, with the simultaneous engagement of both the researcher and the study participants (Hand, 2003). The realities are multiple and defined variously; further, using Constructivist Grounded Theory helped us to understand these shared realities of the researcher and the participants in exploring what Forum Theater does to an individual. The use of an interactive method of constant comparison, simultaneous data collection, and analysis helped to understand shared multiple realities. As prescribed by Charmaz, the data analysis focused on the interactive characters, comparison, and use of abductive reasoning (Chamraz, 2010). Abductive reasoning was achieved by linking theoretical explanations and finding verification of those explanations through more discussions and interviews until saturation was reached in determining the plausible explanations.

### Ethics

The ethical clearance for the study was obtained from the Institutional Review Committee of the Kathmandu Medical College and Teaching Hospital, Kathmandu, Nepal. Participation in this study was voluntary, and written consent was obtained from each study participant after describing the objective of the study. Verbal consent was obtained for the audio recording of the interviews and group discussions. The discussions and the interviews were conducted by assigning codes to the participants, which helped to maintain anonymity. All the study participants were above 18 years of age. A separate male and female focus group discussions helped to reduce bias and for the adequate voice representation of both sexes. Having separate focus group discussions also facilitated participants to speak more freely on sensitive issues. The focus group sessions moderated by the local (first author) with a western researcher (last author) as an observer helped to reduce the power gradient. The study participants considered the first author as one of them due to his constant engagement with the study participants during data collections and periodic Forum Theater interventions. The female study participants felt that the discussions were itself empowering as it helped them to explore and share personal experiences on various social issues, proving that the study process followed utilitarian ethics. The discussion on the subjects for the next Forum Theater and the sharing of research findings allowed the study participants greater control and engagement, making both the research and data collection process participatory. This helped to reduce the boundaries of the researcher and the researched, with both focusing on taking initiatives for increased social awareness.

## Findings

The section below describes the participants’ perception and understanding of the method, learning, relevance, and impacts made by the Forum Theater interventions. The findings explain the study and seek to add to the existing body of knowledge on how participatory Forum Theater has motivated the audience to engage in individual and collective actions.

**Figure 1.**
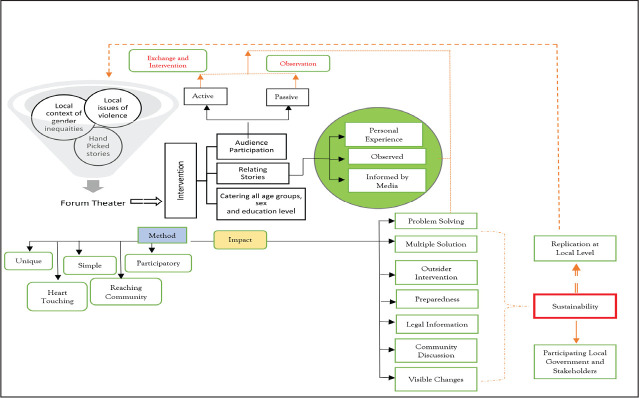
Forum Theater Intervention Process, Perception, and Influence

Figure 1 provides an overview of the Forum Theater intervention process. The figure depicts preparatory works undertaken for the development of Forum Theater interventions and the perception of the receiving audience on the used method. The figure also highlights the engagement of the audience and outlines involved learning processes. The figure lists a few impacts as an outcome of the interventions and shows the process of locally established linkages.

### Heart Touching Method

The Forum Theater performance was witnessed by men, women, children, youths, and the elderly. The shows were organized on the day of the weekly market, which attracted a large number of audiences together with the targeted communities. The audience immediately got attached to the joker, who made the audience participate in playing games and exercises at the beginning of each show. This rapport building with the audience provided them ease to participate later during the interventions and exchanges. The audience cheered, hooted, burst out in laughter, shed tears, and were emotionally involved during each show. The focus group and interview participants made an impression that the theater performance intersected their social realities with an artistic touch, using language that touched their hearts. The Forum Theater performance was received as simple, clear, and understandable to all audiences. A female focus group discussion participant expressed, “The Forum Theater was good; it was easy to follow and understand. The performance was simple and everyone including the illiterates could understand.”

The participants felt a closer connection and developed an emotional involvement with the performing characters. The audiences were seen crying, sobbing, and shedding tears when characters performed on scenes of violence, like when a female character was violently beaten by her husband, or during the scene when a character cried out with grief over her lost mother. The audiences were in rage during the scene when the characters performed the role of angry neighbors gathered to beat an elderly woman accused of witchcraft. The audiences were also seen laughing and cheering during skits showing humor and satire. The participants felt that live performances of Forum Theater provided opportunities for learning as they developed empathy and allowed the audience to imagine themselves going through what a character shows through a performance.

The focus group and interview participants believed that the performances were not only entertaining but also increased their learning. The participants felt that most of the methods used for increasing awareness were either entertaining or educating, but the combination of both, “edutainment” was only found in this method. One of the female participants, individual interview, felt that the method used was more effective. She stated, “Compared to workshops and training on gender issues performed inside a closed room, the Forum Theater method conducted in open-air excites more people and they participate willingly to increase their knowledge.”

For a few of the interview participants, the Forum Theater performances had been the very first exposure to any kind of awareness-raising reaching their villages. The participants were thankful for the opportunity, which helped to increase their knowledge. A male interview participant adds, “This method of informing people with the use of interactive theater methods touches the hearts of the people.”

### Learning, Observing, and Doing

It was learned from the discussion that it was not necessary to be an active audience to learn. The discussion echoed that learning through observation in identifying oppressive situations and developing an understanding of negotiation was identified as the learning method for non-participating audiences. A female focus group participant reflects, “This method enriches all audiences. The actively engaging audiences learn through their engagement while a passive audience who do not come at the front collects information on what to do in similar situations.”

During the performance, it was observed that not all audiences were vocal and got actively engaged during the exchanges and interventions. The discussion participants felt that the shy audience was hesitant to participate, and they considered this perfectly natural. However, the participant had a firm belief that passive audiences were also able to grasp the delivered message. One of the female discussion participants clarifies, “Although not everyone stepped in during the intervention for the discussion, there were many people in the audience giving their opinions to those sitting nearby. How could people speak if they did not get the messages?”

The participants felt that the Forum Theater method was unique as it asked for direct engagement and participation of the audience to try solving different problems. They perceived that the method engaged the audience to think and act through dialogues and negotiations to find solutions. The participants felt that the essence of the Forum Theater was to make the audience grasp the idea that multiple solutions were available for a single problem. They felt the method provided an opportunity to try out recommendations and observe its consequences. One of the male interview participants stated, “Forum Theater makes the community more responsible for their problem. It has helped the community to realize that they should have the capacity for such problem-solving with multiple possibilities.”

The Forum Theater performance was video recorded by several audiences using their cell phones. The participants explained during the data collection that the recording allowed them to watch the performance later and share it along with their family and friends who were not present during the shows. One of the female interview participants stated, “We had recorded the shows and showed them to other people who could not join us for the show. I also showed the recording at my parental home in a different village.”

### Witnessing Realities

The audience perceived the Forum Theater performance as reality rather than the performance of mere fictitious stories. The audience expressed an immediate connection with the storyline, as it reflected situations and events happening around them in their daily lives, to which they could easily relate. The participants felt that the stories were about the real events occurring in their community, which they had seen, experienced, or heard. One of the female participants in the focus group discussion clarified,

I was amongst some elderly people in the audience watching the show, and I could overhear them discussing the plots referring to the incidents that they had witnessed in the village, and at times they would even foretell what would happen and it came true later in the performance.

The participants described incidences of violence against women and inequalities occurring in their communities. The participants reported that inequalities faced by women in the name of witchcraft, alcohol-related violence, and gambling habits of men, and sex trafficking in the name of foreign employment as shown in the Forum Theater were sighted in their community. One of the male participants in the interview suggested as follows:

Yes, the story shown in the forum theater is true. Some people get drunk and fight, people are wasting their money and time on gambling, and eve-teasing and bullying exist in schools. The story feels real to our setting.

The participants disclosed that sexual violence occurred in the community, but they rarely heard about it. The participants inferred that underreporting occurred because reporting of sexual violence shuns the victim’s family and speaking about sex issues was still considered as a taboo and a private affair in the community. The participants also informed that people with higher social status and money were mostly involved in sexual violence, and they utilized their power and money to silence the victims. One of the male interview participants concludes:

People have related sexual violence with their family status and tend not to report it as it disgraces the family. I feel that sexual violence as seen in the performance is on the rise due to underreporting and due to the increasing use of technology and access to digital devices.

The participant informed that the trends of foreign migration were on the rise in the community. The discussions highlighted that almost every single household in the community had one absent male. It also became evident from the discussions that women’s engagement in foreign employment increased their risk of sex trafficking. One of the male focus group participants adds, “Despite knowing that there is victimization at each step of foreign employment, women have been falling into its trap.”

### Increased Ability

The participants believed that the Forum Theater provided information and abilities to work both individually and collectively to solve problems. They felt that witnessing the shows made them more aware of how the problem arises and informed them of ways to overcome it. The participants felt that the take-home message from the show was the use of participatory methods in identifying a problem and for finding a solution with dialogues. One of the female discussion participants stated, “The Forum Theater taught us about the strength of participation and has increased our capacity. It has also made us realize the need for self-responsibility in solving problems that are encountered.”

Citing an example of participation from the Forum Theater performance, one of the individual interview participants was convinced that this approach for problem-solving, with community engagement and working on different possibilities, could help them tackle most of their problems. She stated:

It was clear when an elderly woman participating in the Forum Theater said that ending violence has to be an issue involving the community. This facilitation is needed to end violence and victimization, and if it does not work, the legal process has to follow.

The performances were also perceived by the study participants as a platform for receiving information on legal and various unacceptable behaviors, indicative of gender inequality. The Forum Theater also involved the participation of police, local leaders, and representatives of local groups during the exchange and intervention. This helped the audience to get information on various laws, legal procedures, and services available at the local level. The participants felt that information as such was extremely helpful as most of the people were unaware of processes and methods after an incidence of violence. A female interview participant adds,

I have watched all the forum theater shows and I have heard people discussing and relating the stories of alcohol abuse, witchcraft, and other common violence. Women have started resisting males under the influence of alcohol and this is a visible change.

### Dialogue for Change

Active participation or just witnessing of the Forum Theater made the audience believe that dialogue is essential for ending the *status quo* of oppression and abusive behavior. The Forum Theater performance, together with community participation, was a new and engaging experience for the community. One of the male interview participants suggests that the performances had helped the audience to become more engaged in issues important to them. He adds, “It is not just me, but most of us believe that this participatory method is engaging. People can speak their minds, and this is rewarding.”

The participants have developed an understanding that being a silent sufferer not only increased vulnerability toward victimization but also provided strength to the perpetrator in continuing his/her oppressive actions. Giving an example of the impact of the Forum Theater performance, a female interviewee informed that women had started questioning and protesting against abuse and seeking justice through communal engagements. She stated,

There was a recent event in the village where a woman faced severe abuse from the family. We came together as a community in support of victim women and with the help from the police; we helped the women to get justice.

Another female interviewee emphasized the need to stop being silent, stating the following:

The issue of violence against women is not new; we have been facing it for a very long. Now, I feel we have to speak against it. Staying silent and not protesting is also the reason why violence is on the rise.

It became evident from the discussions and interviews that community discussions among males and females on local issues of inequalities, violence, and sex trafficking had started after the performances, something that was non-existent earlier. The discussions and exchange of thoughts on the causes and possible solutions had sparked a communal dialogue. A female focus group participant stated,

The most noticeable changes are that people have started speaking. Earlier, they used to be silent and feel shy and hesitant even for discussing things important to them but now we have started discussing publicly.

The Forum Theater was identified as instilling changes as the participation enabled the audience to think in new ways. A sense of obligation was developing among those who had participated in responding to oppressive behaviors. A female focus group participant felt that engagement in the Forum Theater had brought about enormous changes in her, and she was committed to getting further involved in bringing about changes. She stated,

I see changes in myself after my participation in the Forum Theater. I have begun questioning myself that our society is laden with so many inequalities and should not I be responsible to make it better. We should all work together in creating better life opportunities for all.

### Increased Ability

**Figure 2.**
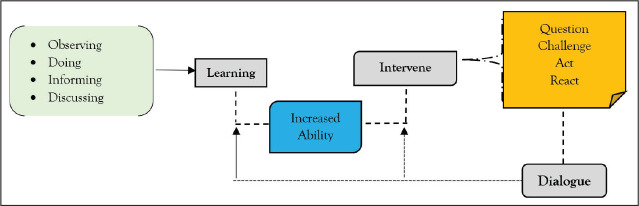
Forum Theater Transforming an Individual.

Figure 2 provides an overview of the changes perceived at an individual level after the exposure to the Forum Theater. It indicates that learning from both active and passive engagement provides opportunities to act, observe, inform, and discuss. The learning does not necessarily require active engagement with observation itself being a process of information collection and knowledge building. This learning helps to overcome internalized discriminatory values, beliefs, and attitudes that are conditioned to various norms and practices. Moreover, learning through active engagement and observation has informed the audience on legal aspects of violence, complaint filing process, punishments, and available communal support. This has allowed the audience to increase their preparedness identified as the increased ability. This change in ability empowers an individual with knowledge, empathy, and motivation to intervene during unfair and unjust situations. The changes are apparent when the informed audience acts for self-help or reaches out to help others using dialogue. This dialogue and negotiation capacitate individuals to resist and challenge oppression. Further dialogue, resistance, and negotiation help to identify possible multiple solutions. A loop emerges as involvement with dialogue facilitates more learning, more ability, and more preparedness for interventions required in ending oppressive situations.

## Discussion

The discussion and interviews with the study respondents infer that participatory and embodied learning can be an effective tool for developing a change to reduce gender inequalities and violence against women. The study relied on face-to-face discussions using focus groups and individual interviews. This helped to reduce recall bias and provided insights on the relevance, perception, effectiveness, and participant’s understanding of the Forum Theater shows. ##This study finds that learning processes with dialogue help to develop critical thinking; show empathy toward the victim; challenge existing behaviors, beliefs, and norms; and motivate the audience to take prompt required actions. The study finds that the audience’s engagement has provided opportunities to safely rehearse the unjust real-life situations of oppression encountered in daily lives.

The study reached both male and female study participants in 10 different villages. The study participants for the focus group consisted of the dominant ethnic community inhabiting the study area while the individual interviews included participants from different ethnicity and caste background such as Tharus, Rajbanshi, Dhimal, Madheshi, Bhramin, Chetrris, Janajati, and Dalit. Despite language diversity, all participants involved in the study were comfortable establishing fluent communication in the national Nepali language; hence, the use of a translator was not required. The findings from this study represent the voice of heterogeneous sociocultural settings and the findings can be transferred to similar settings. However, the issues raised by the study to establishing gender equality, reducing violence, and increased capacity of the individuals are universal and can be relevant elsewhere. The study caters to a global audience through immersion in the participatory methods outlined in the study with possibilities of replication adapting to the local context.

The earlier research utilizing participatory theater has produced mixed results but mostly with encouraging findings on participation and the transformations achieved through it. A study utilizing Forum Theater for bystanders for gender violence prevention in the US has identified that Forum Theater use has increased awareness, developed intervention skills, and aided in developing community responsibilities for violence prevention (Mitchell & Freitag, 2011). The findings from this study suggest that awareness-raising through Forum Theater empowers communities with ideas leading to action, which is the primary step required for any social transformation. Earlier works have equally stressed the importance of participatory methods as empowering tools for social engagement as they help to identify and define problems, provide support to develop critical reflections on the problem, and offer guidance in finding answers (Friere, 1970; Hooks, 1994).

A study on participatory theater identifies that theater not only connects people with emotions and promoting awareness, but it also raises communal knowledge, to ward off discriminatory practices arising from prevailing norms (Daruwalla et al., 2019). The theoretical concept of increased ability developed by this study indicates that information and knowledge gained through active engagements provide greater learning, motivating individuals to challenge and intervene and to bring about change also for others. The study finds that knowledge gained through participation has enabled the audience to challenge and question stereotypes, behaviors, and norms; furthermore, the engagement with dialogue has produced change. Similarly, a previous study on Forum Theater has identified that individual behaviors are often restricted by structural discriminations, and changes are possible with collective actions in dismantling oppressive/limiting structures (Wrentschur & Moser, 2014). A previous study on Forum Theater to address gender issues highlights these integrated efforts to change, which are rooted in power relations, structures, and hierarchies (Österlind, 2008). Moreover, Forum Theater use has been identified to instill this idea of possible changes with the culmination of realization that changes are possible and that the power to control one’s life remains with them (Thomson & Wood, 2001).

A study conducted in Iran on gender performance and identity creation has identified theater as a practice of self-representation that has helped to transform participants into an active audience (Lev-Aladgem & First, 2004). The findings from this study reveal that changes can occur with reflections and by challenging oppressive behaviors with dialogues aiming for possible transformations. Furthermore, the promotion of gender equality for violence prevention has emphasized the need for individual and social engagement to address gender norms, and for interventions that help change attitudes (WHO, 2009) similar to engaging Forum Theater used by the study. The use of engaging and participatory approaches advocates for the development of agency in addressing gender differences and unequal power relationships (Haberland, 2015). The development of this self-agency and motivation to negotiate for equating power relationships can be acquired through engagement to increase ability as found by this study.

The initiation of social discussions as reported by this study increases ownership in the community and equips them with additional information, beliefs, and skills required for required future interventions (Yoshihama & Tolman, 2015). The use of narrative theater to decrease domestic violence in the Ugandan refugee setting has identified that theater engagements possess the potential to initiate transformations using local knowledge (Sliep et al., 2004). The study also identifies that participatory engagement has helped participants to explore social realities and develop abilities to overcoming challenges with required collective actions. Individual behaviors and attitudes are fluid, and interventions like these can help one to overcome, reduce, or speak against oppression. The social processes and structures are also a co-creation of the collective actions, and the proactive engagement enables individuals and the community to aim for ending oppressive real-life situations.

Earlier research has stressed that interactive and participatory methods lead to more effective learning as these methods help to grasp insights with reflective learning and problem solving relying on personal experiences (Brankovic, 2019; Gibbs et al., 2019; Grey et al., 1999; Margalit et al., 2004; Milhausen & Herold, 1999; Ríos & Sánchez-Sosa, 2002). The participants for this study have reflected on Forum Theater as a unique participatory method for developing critical views on existing discriminatory gender practices. Earlier evaluations of interactive methods also identify that participatory processes enable the participants to re-evaluate their beliefs, and develop critical reasoning for required action (Conrad, 2004; Heras & Tàbara, 2014; Quinn, 2014). Forum Theater on domestic violence has been tested in Taiwan and the study identifies that the engagement with Forum Theater enables the local audience and encourages them to provide alternatives for solving domestic conflicts (Wang, 2010) similar to the possibilities of multiple solutions identified through the intervention in this study.

The enormity of problems emerging from gender inequality and violence against women is not new for Nepal; the gender hierarchy and the male-centered power relations have been cited as reasons for inequalities and violence (Dahal et al., 2019; UNDP, 2014). In this background, it is important to have simulations with Forum Theater rehearsals for real life to engage individuals and the community to address discrimination and violence. The engaging audience is more aware of the root causes of exploitation and abuse, allowing them to identify oppression with considerable ease. The Forum Theater opens spaces for these people to assemble and discuss oppression and its end.

We learned from the interviews that one of the communities had replicated the practice of Forum Theater. It was reported that the community organized themselves, developed a play, and performed it to find a solution for the local sanitation issues. This occurred after the second Forum Theater performance. Specifically, when the team reached the site for the final round of performance, the first author had an opportunity to personally communicate with the local mayor. The mayor, who had received information about the community Forum Theater performance, thanked the study for introducing a powerful tool for civic engagement. The discussions and interviews made us aware that the message of Forum Theater intervention had been diffused widely. The video recording of the shows had reached other places through personal sharing and social media. It is too early to discuss sustainability but understanding that the practice has been replicated informs of budding ownership and ushers hopes of sustenance. Likewise, the established linkages with other stakeholders produce vibrant faith in continued efforts.

## Strengths and Limitations

The study was focused on process evaluation using the exploratory method. The study identifies participants’ perception and understanding of the method, learning, relevance, and impacts made by the Forum Theater interventions. The study explored how participatory Forum Theater has motivated the audience to engage in individual and collective actions. The study relied on continued discussions among researchers and the theater partner from the beginning. The developed guides consisted of open-ended questions to reduce any possible bias. The study relied on conducting separate male and female focus groups and conducted interviews in private to promote self-disclosure. The preliminary findings from the data analysis were shared with the focus group participants to help increase respondent validation. The multistage focus group method also aided to triangulate the collected information. The discussion among the researchers at each level of the data analysis contributed to consistency and helped to bring forward any overlooked information. The self-reported findings on changes in individual behaviors of the study participants have not been further evaluated. Any intervention-based research on awareness can be challenging as behavioral changes are difficult to assess. Social desirability might influence the study which requires greater scrutiny for the research process. Changes are only sustainable with committed ownership; future research should aim to facilitate the community to sketch the process of awareness and information-based activities. The study recognizes that focus groups and interviews are guided by social context and influences. A post-evaluation study to assess behavioral and social changes would have helped to measure the sustenance of the Forum Theater interventions.

## Study Implications

The study has contributed with new knowledge on participatory methods of community engagement, utilizing the Forum Theater intervention. The theoretical model developed from the study can help future research on establishing the efficacy of Forum Theater, while the intervention and evaluation methods can be replicated elsewhere. The study findings on change at an individual level and the collective action at the community level for establishing equality and reducing violence can be useful for practitioners and other relevant stakeholders. Participatory research methods can also help develop tools for assessing inequality and violence. The use of participatory methods through constant engagement such as during the script development, use of reflective dialogues thorough interviews and discussions to share perceptions and realities and participatory process evaluation increases ownership and aims for the sustenance of the efforts. This study has captured both activism and research practiced by the practitioners and the academicians, providing insights into both the involved processes. The study can equally benefit the policymakers and other political actors for encouraging participatory methods for developing agency, voice, and activities directed toward producing social transformations. The findings from the study encourage further testing of the participatory Forum Theater for drawing more causal inferences.

## Author Biographies

**Pranab Dahal**, PhD, MSc, MA, is a researcher at the Department of Health Sciences at Linnaeus University, Sweden. His research interest is on gender inequality, violence, sex-trafficking, and intervention-based community projects.

**Sunil Kumar Joshi** is a medical doctor (MD) specialized in Occupational Medicine, Diabetology, and PhD in Public Health and Community Medicine. He is working as the head of the department of Community Medicine at Kathmandu Medical College, Kathmandu University, Nepal. Also, he is a visiting professor at the University of the West of England, Bristol, United Kingdom. Professor Joshi has assisted the Nepal Government, Ministry of Health and Population in formulating policies, strategies, and action plans in injuries, violence, and disability prevention.

**Katarina Swahnberg** is an RN, educated at the Red Cross Nursing School, Stockholm. She has a BSc in International Health Care, and a BSc and an MSc in Public Health. She defended her PhD thesis on violence against women and abuse in health care in 2003. She is a professor in Health Sciences and Global Health at the Department of Health and Caring Sciences, Linnaeus University, Kalmar, Sweden.
